# Detection of Tuberculosis Recurrence, Diagnosis and Treatment Response by a Blood Transcriptomic Risk Signature in HIV-Infected Persons on Antiretroviral Therapy

**DOI:** 10.3389/fmicb.2019.01441

**Published:** 2019-06-26

**Authors:** Fatoumatta Darboe, Stanley Kimbung Mbandi, Kogieleum Naidoo, Nonhlanhla Yende-Zuma, Lara Lewis, Ethan G. Thompson, Fergal J. Duffy, Michelle Fisher, Elizabeth Filander, Michele van Rooyen, Nicole Bilek, Simbarashe Mabwe, Lyle R. McKinnon, Novel Chegou, Andre Loxton, Gerhard Walzl, Gerard Tromp, Nesri Padayatchi, Dhineshree Govender, Mark Hatherill, Salim Abdool Karim, Daniel E. Zak, Adam Penn-Nicholson, Thomas J. Scriba, The SATVI Clinical Immunology Team

**Affiliations:** ^1^South African Tuberculosis Vaccine Initiative, Institute of Infectious Disease and Molecular Medicine and Division of Immunology and Department of Pathology, University of Cape Town, Cape Town, South Africa; ^2^Centre for the AIDS Programme of Research in South Africa (CAPRISA), Durban, South Africa; ^3^MRC-CAPRISA HIV-TB Pathogenesis and Treatment Research Unit, Doris Duke Medical Research Institute, University of KwaZulu-Natal, Durban, South Africa; ^4^Center for Infectious Disease Research, Seattle, WA, United States; ^5^Department of Medical Microbiology and Infectious Diseases, University of Manitoba, Winnipeg, MB, Canada; ^6^DST-NRF Centre of Excellence for Biomedical TB Research and South African Medical Research Council Centre for TB Research, Division of Molecular Biology and Human Genetics, Faculty of Medicine and Health Sciences, Stellenbosch University, Tygerberg, South Africa; ^7^South African Tuberculosis Bioinformatics Initiative (SATBBI), Division of Molecular Biology and Human Genetics, Faculty of Medicine and Heath Sciences, Stellenbosch University, Tygerberg, South Africa; ^8^Department of Epidemiology, Columbia University, New York, NY, United States

**Keywords:** tuberculosis, recurrence, diagnosis, treatment, transcriptomic signature, HIV, antiretroviral therapy

## Abstract

HIV-infected individuals are at high risk of tuberculosis disease and those with prior tuberculosis episodes are at even higher risk of disease recurrence. A non-sputum biomarker that identifies individuals at highest tuberculosis risk would allow targeted microbiological testing and appropriate treatment and also guide need for prolonged therapy. We determined the utility of a previously developed whole blood transcriptomic correlate of risk (COR) signature for (1) predicting incident recurrent tuberculosis, (2) tuberculosis diagnosis and (3) its potential utility for tuberculosis treatment monitoring in HIV-infected individuals. We retrieved cryopreserved blood specimens from three previously completed clinical studies and measured the COR signature by quantitative microfluidic real-time-PCR. The signature differentiated recurrent tuberculosis progressors from non-progressors within 3 months of diagnosis with an area under the Receiver-operating characteristic (ROC) curve (AUC) of 0.72 (95% confidence interval (CI), 0.58–0.85) amongst HIV-infected individuals on antiretroviral therapy (ART). Twenty-five of 43 progressors (58%) were asymptomatic at microbiological diagnosis and thus had subclinical disease. The signature showed excellent diagnostic discrimination between HIV-uninfected tuberculosis cases and controls (AUC 0.97; 95%CI 0.94–1). Performance was lower in HIV-infected individuals (AUC 0.83; 95%CI 0.81–0.96) and signature scores were directly associated with HIV viral loads. Tuberculosis treatment response in HIV-infected individuals on ART with a new recurrent tuberculosis diagnosis was also assessed. Signature scores decreased significantly during treatment. However, pre-treatment scores could not differentiate between those who became sputum negative before and after 2 months. Direct application of the unmodified blood transcriptomic COR signature detected subclinical and active tuberculosis by blind validation in HIV-infected individuals. However, prognostic performance for recurrent tuberculosis, and performance as diagnostic and as treatment monitoring tool in HIV-infected persons was inferior to published results from HIV-negative cohorts. Our results suggest that performance of transcriptomic signatures comprising interferon stimulated genes are negatively affected in HIV-infected individuals, especially in those with incompletely suppressed viral loads.

## Introduction

Every year more than 10 million people develop tuberculosis (TB) disease, the leading infectious cause of death (World Health Organization [WHO], 2017b). Individuals who have had TB previously are at significantly increased risk of developing recurrent disease. Incidence of recurrence within the first year following completion of TB treatment is 2–8% (Friedrich et al., 2013; Gillespie et al., 2014; Merle et al., 2014), which is several-fold higher than the rate of incident TB disease in the same populations with no history of TB. Recurrent TB disease thus contributes substantially to the significant disease burden in settings where TB is endemic. A further compounding factor is underlying HIV infection, which not only increases the risk of TB in general, but further increases risk of recurrent TB (Korenromp et al., 2003; Maher et al., 2005). For example, risk of recurrent TB disease was five times higher in HIV-infected South African gold miners relative to their HIV-uninfected colleagues (Mallory et al., 2000). Early and accurate identification of HIV-infected individuals who are at high risk of recurrent TB would allow targeted investigation for disease, facilitating earlier provision of antibiotic treatment.

Several studies have shown that blood transcriptomic signatures can accurately differentiate between active TB disease, asymptomatic *Mycobacterium tuberculosis* (*Mtb*) infection and other respiratory diseases such as pneumonia and sarcoidosis, demonstrating promising diagnostic potential of immune gene expression in predicting TB (Berry et al., 2010; Maertzdorf et al., 2012; Bloom et al., 2013; Kaforou et al., 2013; Anderson et al., 2014; Sweeney et al., 2016). It is likely that such transcriptomic signatures, which detect inflammatory signals such as elevated interferon stimulated gene (ISG) expression commonly associated with TB disease, would also have promising utility for diagnosing recurrent TB, but to our knowledge this has yet to be tested.

We recently developed and validated a whole blood transcriptomic correlate of risk signature [Adolescent Cohort Study (ACS) COR] for TB comprising 16 ISGs, which showed prognostic value for incident TB disease in adults and adolescents from two African countries (Zak et al., 2016). Analysis of the inflammatory processes underlying TB disease progression strongly suggests that the COR signature, as well as other similar transcriptomic signatures, detect incipient or subclinical disease weeks to months before the manifestation of clinical symptoms (Scriba et al., 2017; Suliman et al., 2018). We subsequently reduced the COR signature to an 11-gene signature to improve laboratory throughput, with no effect on diagnostic or prognostic performance (Darboe et al., 2018). Despite strong ISG expression in granulocytes (Berry et al., 2010; Scriba et al., 2017), diagnostic performance of the signature in HIV-uninfected adults was not different in cryopreserved PBMC and matched whole blood (Darboe et al., 2018). This opened the possibility to evaluate the COR signature in rare longitudinal cohorts from whom only biobanked PBMCs are available. In this study we aimed to determine the prognostic value of this 11-gene COR signature for recurrent TB in people living with HIV who participated in the “TB Recurrence upon Treatment with HAART” (TRuTH) study conducted at the Centre for the Aids Programme of Research In South Africa (CAPRISA) in Durban, South Africa (Maharaj et al., 2017; Sivro et al., 2017).

In addition, we aimed to evaluate the utility of the signature for monitoring treatment response in HIV-infected patients treated for recurrent TB disease. Standard treatment of drug-sensitive TB lasts at least 6 months and commonly comprises a regimen of rifampicin, isoniazid, pyrazinamide, and ethambutol during the first 2 months followed by isoniazid and rifampicin for 4 months. Although this strategy has a high success rate resulting in bacteriological cure in approximately 86% under routine, programmatic field conditions (World Health Organization [WHO], 2017b), treatment failure and relapse are a very significant problem. Treatment success is typically monitored by detecting sputum conversion (conversion to a negative bacteriological test), measured by *Mtb* culture. However, 2-month sputum culture conversion has a low predictive value for predicting treatment failure and relapse (Horne et al., 2010). Clinical trials of short-course TB therapy regimen report high relapse rates in participants despite high culture conversion rates after 2 months of therapy (Burman et al., 2006; Gillespie et al., 2014; Merle et al., 2014). Novel, non-sputum-based tests that can accurately monitor treatment efficacy, detect treatment failure and predict relapse are urgently required. Several studies have shown that blood transcriptomic signatures can be used to monitor TB treatment since they can indicate resolution of TB-associated inflammation and ISG expression levels (Berry et al., 2010; Bloom et al., 2012, 2013; Cliff et al., 2013; Sweeney et al., 2016). In a recent study of 99 HIV-uninfected drug-sensitive TB cases on TB treatment, we showed that the ACS COR signature, measured at treatment baseline, could differentiate between patients with cure and treatment failure and could predict treatment outcome (Thompson et al., 2017). It is currently not known if blood transcriptomic signatures will similarly, indicate resolution of TB-associated inflammation in HIV-infected persons or how a previous episode(s) of TB affects the kinetics of such signatures during treatment. In the second part of the study, we determined the utility of the 11-gene ACS COR signature for monitoring HIV-infected patients treated for recurrent TB disease as part of the IMPRESS trial, conducted at CAPRISA in Durban, South Africa (Naidoo et al., 2017).

## Materials and Methods

### Study Populations

Samples collected from participants enrolled into three previously completed independent clinical studies were included.

#### Prognostic Cohort

The first was the TB Recurrence upon Treatment with HAART (TRuTH) CAPRISA 005 study (Maharaj et al., 2017; Sivro et al., 2017). This prospective cohort study, conducted between 2009 and 2013, aimed to assess risk factors associated with progression to recurrent TB disease during a 3-year longitudinal follow up in HIV-infected persons who began ART during treatment of the previous TB episode. At study enrolment and 3-monthly thereafter, participants were assessed for recurrent TB, and peripheral blood mononuclear cells (PBMC) cryopreserved. Investigation for TB included both sputum induction for liquid culture and assessment of clinical symptoms of TB disease. Additional sputum cultures and clinical investigations were performed when participants presented with TB symptoms or abnormal chest x-rays outside of follow-up time points. Participants who developed microbiologically-confirmed recurrent TB during follow up were labeled as progressors, and selected for further analysis. For each progressor we selected two non-progressors, who remained TB-free during follow-up, matching for gender and date (to within a 90-day window) of ART initiation. Time to recurrent TB disease was calculated for progressors by subtracting the sample collection date from the date of recurrent TB diagnosis. For samples from non-progressors the “time to recurrent TB” derived from their respective matched progressor, as shown in Figure 2A,B, was assigned. This “time to recurrent TB” was used where necessary for comparative longitudinal analyses between progressors and non-progressors, even though non-progressors did not develop disease.

#### Treatment Response Cohort

The second study was the open-label, randomized controlled trial, “Improving Retreatment Success” (IMPRESS) (Naidoo et al., 2017). This trial was designed to determine if a moxifloxacin-containing 24-week regimen, in which moxifloxacin was substituted for ethambutol, would improve TB retreatment outcomes relative to the standard TB treatment regimen. The study started enrolment in November 2013 in Durban, KwaZulu-Natal, and follow-up ended in July 2017. The trial enrolled adults with a previous history of TB disease who received a new diagnosis of drug-sensitive TB by positive Xpert MTB/RIF (Cepheid) or sputum smear or both. Participants attended two-weekly visits during the intensive phase of treatment and monthly visits during the continuous phase. Sputum samples were collected for culture testing every 2 weeks during the intensive phase of treatment and monthly thereafter until successful treatment completion. Whole blood was collected in PAXgene tubes (Qiagen) at baseline, 2, 6, 8, and 14 months after start of TB treatment. Inclusion of PAXgene tube collection was initiated after the start of the trial and therefore some participants did not have PAXgene blood collected at all timepoints. Only those with at least two PAXgene samples collected during TB treatment were included in transcriptomic analyses. “Time to culture conversion” was calculated by subtracting the date of the first positive MGIT culture (BD Biosciences) result at diagnosis from the date of the first persistent negative culture result and this “time to culture conversion” was used to stratify patients into early (conversion within 8 weeks) and late (conversion after 8 weeks) converters.

#### Diagnostic Cohort

In the third study, the Cross-sectional TB cohort (CTBC), we assessed the impact of HIV-infection on the diagnostic performance of the 11-gene ACS COR signature in an observational study of HIV-infected (*n* = 40) and uninfected (*n* = 60) South African adults from the Worcester region, Western Cape. The study was designed to determine if the COR signature could distinguish between *Mtb* infection and active TB disease, diagnosed by a positive sputum Xpert MTB/RIF test. *Mtb* infection was diagnosed by a positive QuantiFERON TB Gold in-tube assay (Qiagen; cut-off >0.35 IU/mL). HIV-infection was diagnosed with the Determine HIV1/2 test (Alere). Whole blood was collected in PAXgene tubes and PBMC were isolated from heparinized blood collected in Cell Preparation Tubes (BD Biosciences). All participants provided written, informed consent. The study protocol for the CTBC diagnostic study was reviewed and approved by the Human Research Ethics Committee of the University of Cape Town (HREC 761/2015).

### Ethics Statement

#### Prognostic Cohort

All participants of the previously completed TB Recurrence upon Treatment with HAART (TRuTH) CAPRISA 005 study (ClinicalTrials.gov, NCT01539005; SANCTR DOH-27-0909-3040) provided written, informed consent in accordance with the Declaration of Helsinki (Maharaj et al., 2017; Sivro et al., 2017). The study protocol for TRuTH was reviewed and approved by the University of Kwazulu-Natal Research Ethics Committee (BREC No.: BF051/09).

#### Treatment Response Cohort

The “Improving Retreatment Success” trial (IMPRESS, ClinicalTrials.gov, NTC02114684; SANCTR DOH-27-0414-4576) was approved by the Medicines Control Council of South Africa (MCC Ref:20130510) (Naidoo et al., 2017). All participants provided written, informed consent in accordance with the Declaration of Helsinki, and the study protocol for IMPRESS was reviewed and approved by the University of Kwazulu-Natal Research Ethics Committee (BREC No. BFC029/13).

#### Diagnostic Cohort

The study protocol for the Cross-sectional TB cohort (CTBC) diagnostic study was reviewed and approved by the Human Research Ethics Committee of the University of Cape Town (HREC 761/2015). All participants provided written, informed consent, in accordance with the Declaration of Helsinki.

### RNA Extraction and ACS COR Signature Measurement

RNA was extracted from thawed cryopreserved PBMC samples using the RNEasy plus Mini extraction kit (Qiagen) per manufacturer’s instructions or from PAXgene samples using the SimplyAmp extraction kit (Promega) using the Tecan Freedom EVO 150 automated system. Expression of mRNA transcripts comprising the 11-gene ACS COR signature (Darboe et al., 2018) was measured using commercial TaqMan primer/probe sets (Thermo Fisher Scientific; Supplementary Appendix A1) by microfluidic qRT-PCR using 96.96 Gene Expression chips (Fluidigm) on the Biomark HD multiplex instrument (Fluidigm) after cDNA synthesis using Superscript II reverse transcriptase (Invitrogen), as previously described (Zak et al., 2016). Signature score data for the 11-gene ACS COR signature from the CTBC, TRUTH and IMPRESS cohorts are available in Supplementary Tables S1–S3. Signature scores for the 11-gene ACS COR signature were calculated using in-house scripts written in R as previously described (Zak et al., 2016; Darboe et al., 2018), and provided as an Excel template (Supplementary Tables S4, S5).

### Statistical Analyses

The pROC (Robin et al., 2013) and verification (Pocernich, 2015) R packages were used to calculate area under the receiver operating characteristic (ROC) curve (AUC) and associated 95% confidence intervals (CI), and for comparing AUCs. Medians and 95% CIs of relative differences in mRNA transcript expression between groups (e.g., HIV-infected and uninfected persons) were calculated using the rank inversion method and bootstrapping 2,000 times using the quantreg R package (Koenker, 1994). The Mann-Whitney U test was used to analyze differences between groups. The Wilxocon signed rank test and McNemar tests were used to compare continuous and binary baseline characteristics, respectively, between progressors and non-progressors in the TRuTH cohort. The Mann-Whitney U test and the Chi-Square test were used to compare demographic and clinical characteristics of the early and late converters in the IMPRESS study.

## Results

### Participant Characteristics

#### Prognostic Cohort

**FIGURE 1:**
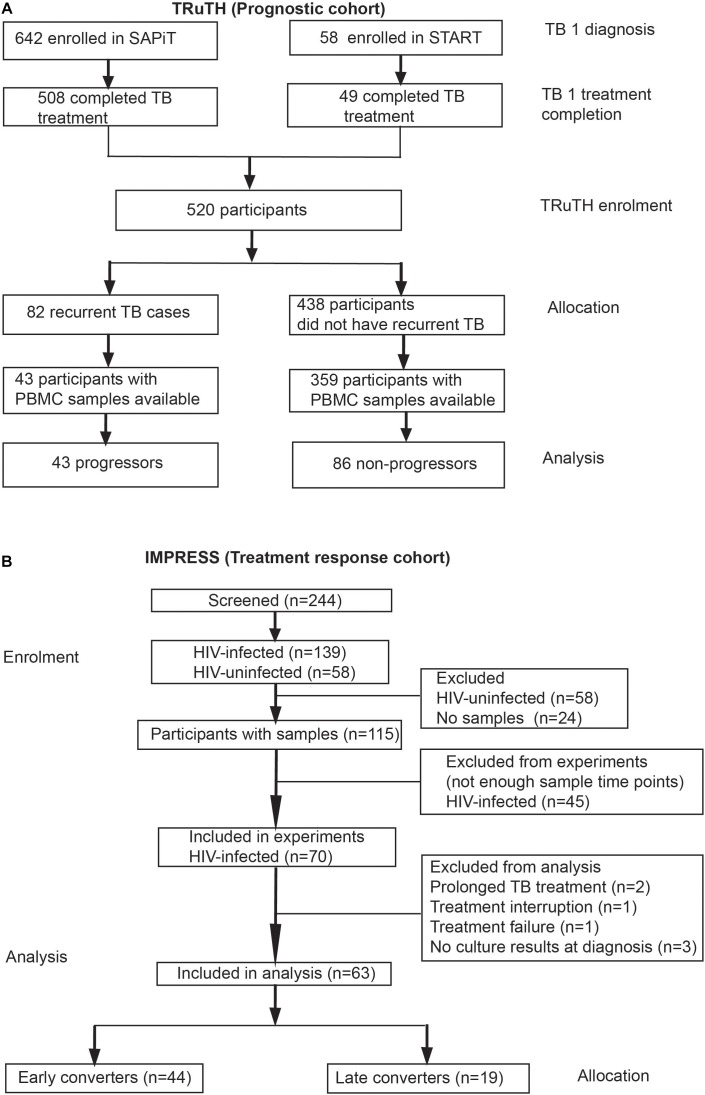
CONSORT of participant enrolment, allocation and analyses in the **(A)** TRuTH and **(B)** IMPRESS cohorts. Participants in the TRuTH study were previously enrolled into either the SAPiT (Abdool Karim et al., 2010; Abdool Karim et al., 2011) or START (Gengiah et al., 2012) studies.

**FIGURE 2:**
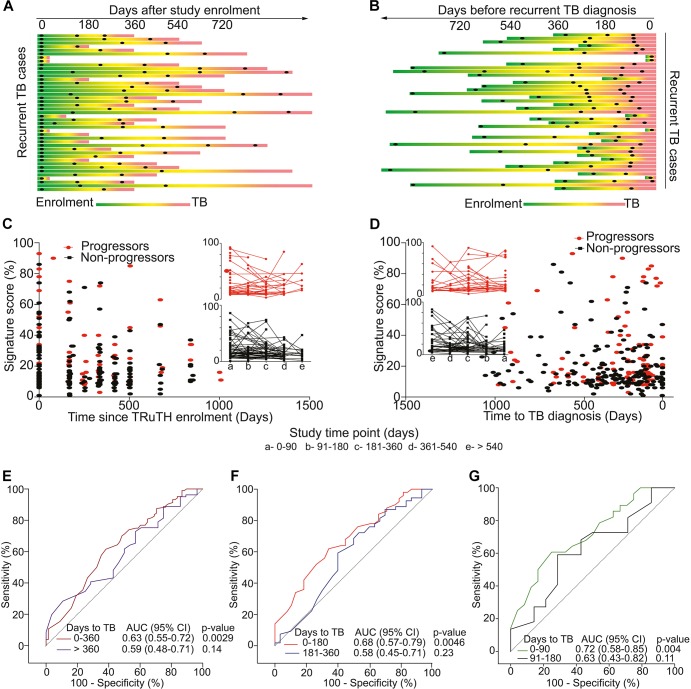
Study design and 11-gene ACS COR signature performance in the TRuTH study. Horizontal lines depict the 43 TRuTH cohort progressors and show PBMC samples (black dots) collected in each individual. **(A)** TRuTH progressors aligned to their time of TRuTH study enrolment. The time of TB diagnosis is indicated by the most left-hand end of each line (in red). **(B)** Re-alignment of TRuTH progressors and their samples according to each individual’s date of TB diagnosis (on the right) to facilitate analyses of changes by “time to recurrent TB.” **(C)** 11-gene ACS COR signature scores at each participant visit for progressors (red) and non-progressors (black), aligned by time since TRuTH enrolment. Inserts show the longitudinal kinetics of these scores for progressors (red, top) and non-progressors (black, bottom). 90-day time windows relative to enrolment are depicted by the letters on the x-axis. **(D)** 11-gene ACS COR signature scores realigned to time to recurrent TB diagnosis. **(E–G)** ROC AUCs depicting prognostic performance of the 11-gene ACS COR signature for recurrent TB, showing discrimination of progressors from non-progressors in the TRuTH cohort. Each curve represents a 1-year **(E)**, 6-month **(F)** or 3-month **(G)** time window prior to recurrent TB diagnosis.

Among 520 participants in the longitudinal TRuTH study, 92 episodes of recurrent TB disease were recorded in 82 participants. However, only 43 participants had sufficient cryopreserved PBMC samples available for inclusion as progressors; 86 non-progressors were selected for our transcriptomic analyses, for a 2:1 control:case matching (Figure 1A). No differences in age and BMI at TRuTH enrolment were observed between progressors and non-progressors (Table 1). Non-progressors had higher median CD4 T-cell counts at baseline compared to progressors [405 vs. 336, respectively (*p* = 0.01)]. A higher proportion of virologically suppressed patients were non-progressors (88.2 vs. 62.8%; *p* < 0.001), most of whom completed TB treatment at an earlier timepoint before TRuTH enrolment than progressors (876 vs. 1079 days; *p* < 0.001). Recurrent TB disease occurred at a median of 470 days (IQR: 250–672 days) after TRuTH enrolment (Figure 2). All available samples from the progressors, collected 6-monthly, were analyzed (Figure 2A), resulting in 103 progressor and 196 non-progressor samples that spanned “time to recurrent TB” between 3 months and >2 years before diagnosis (Figure 2B). CD4 T-cell counts, plasma viral loads (pVL), and body mass index at the start of treatment and end of treatment of the first TB episode are shown in Supplementary Figure S1.

**Table 1:** Demographic and clinical characteristics of progressors and non-progressors in the TRuTH study at study enrolment (prognostic cohort).

Participant characteristics		Progressors (n = 43)	Non-progressors (n = 86)	p-value
Sex, n (%)	Male	21 (49)	42 (49)	1.00
Median age, years (range)		35 (25–53)	37.5 (23–62)	1.00
Race, n (%)	Black African	43 (100)	85 (99)	1.00
	Other	0 (0)	1 (1)	
Median BMI, kg/m^2^ (range)		24 (16.5–41.9)	24.3 (16.4–40.8)	0.58
Previous TB, n (%)	1	27 (62.8)	58 (67.4)	0.56
	2	16 (36.4)	28 (32.6)	
	Median days since previous TB (range)	876 (707–2009)	1079 (706–1866)	<0.001
Median days on ART, (range)^∗^		758 (-22–1988)	775.5 (-28–1803)	0.51
Plasma viral load (pVL), copies/mL^∗∗^	Undetectable, n (%)	35 (81.4)	79 (92.9)	0.01
	Median of log dectectable pVL (range)	4.4 (2.8–5.7)	4.7 (3.8–6.0)	
Median CD4 count (range), cells/μL		336 (14–884)	405 (66–1211)	0.01


*^∗^Two non-progresors started ART after TRUTH enrollment and therefore excluded in this calculation. ^∗∗^Limit of detection of pVL was 400 copies/mL. Only 14 participants had detectable pVL and no formal analysis of pVL magnitude was done.*

#### Treatment Cohort

A total of 197 newly diagnosed active TB cases with a previous history of TB disease were enrolled into the IMPRESS trial (Figure 1B). Most participants were HIV-infected (*n* = 139, 71%). Due to the low number of HIV-uninfected participants with PAXgene samples available, only HIV-infected participants were included in transcriptomic analyses. Among 115 HIV-infected participants with any samples available, 70 participants had PAXgene samples collected at baseline and two additional study time-points and were eligible for inclusion into treatment outcome analyses. Seven participants were excluded due to protocol deviations related to treatment or absence of culture results (Figure 1B), resulting in a final sample size of 63 HIV-infected patients, 44 early converters and 19 late converters (Figure 1B). Age, sex, proportion on anti-retroviral therapy and plasma viral load were not different between late and early converters (Table 2). However, late converters had lower BMI and median CD4 T-cell counts than early converters. A higher proportion of the late converters included in our analyses were in the standard of care arm, i.e., they received ethambutol and not moxifloxacin (*p* = 0.01; Table 2).

**Table 2:** Demographic and clinical characteristics of participants in the IMPRESS study (treatment response cohort).

Participant characteristic		Entire cohort (n = 63)	Early converters (n = 44)	Late converters (n = 19)	p-value
Sex, n (%)	Male	44 (69.8)	28 (63.6)	16 (84.2)	0.10
Median age, years (range)		37 (19–51)	37 (21–51)	35 (19–51)	0.30
Race, n (%)	Black African	62 (98.4)	43 (97.7)	19 (100)	0.51
	Cape Mixed Ancestry	1 (1.6)	1 (2.3)	0 (0)	
Median BMI, kg/m^2^ (range)		20.0 (15.6–37.8)	21.1 (16.6–37.8)	19.4 (15.6–26.1)	0.05
Study treatment arm, n (%)	HRZM (Moxifloxacin)	33 (52.4)	28 (63.6)	5 (26.3)	0.01
On ARV^∗^, n (%)		28 (45.2)	19 (35.2)	9 (50)	0.76
Median CD4 counts^∗∗^, (range)		268 (46–849)	285 (46-849)	236.5 (69–477)	0.05
Plasma viral load ^∗∗∗^	Undetectable, n (%)	27 (45.0)	19 (45.2)	8 (44.4)	0.94
	Median detectable pVL, log copies/mL (range)	4.7 (2.8–6.1)	4.7 (3.3—6.1)	4.3 (2.8–5.6)	0.6


*Limit of plasma viral load detection was set at 400 copies/mL. ^∗^Only 62 participants had data available, early converters (*n* = 44), late converters (*n* = 18). ^∗∗^Only 61 participants had CD4 count information; early converters (*n* = 43), late converters (n = 18). ^∗∗∗^Only 60 participants had plasma viral load data; early converters (n = 42), late converters (*n* = 18).*

#### Diagnostic Cohort

Fifty newly diagnosed active TB cases (20 of whom were HIV-infected) and fifty *Mtb* infected persons (20 of whom were HIV-infected) were enrolled into the CTBC. A higher proportion of the HIV-uninfected active TB cases (73%) were male in comparison to their *Mtb*-infected (27%) counterparts (*p* = 0.0007). *Mtb*-infected HIV-uninfected individuals had a higher BMI (median 30.4 vs. 20.5; *p* < 0.0001) and were older (40.5 vs. 34, *p* = 0.01). No differences in the above characteristics were observed between active TB and *Mtb*-infected controls who were HIV-infected.

### Prognostic Performance of 11-Gene ACS COR Signature for Recurrent TB Disease in HIV-Infected Persons on ART

We first determined the prognostic performance of the 11-gene ACS COR signature for incident recurrent TB disease by stratifying signature measurements into 1-year time windows relative to TB diagnosis. Gene expression was measured using RNA isolated from PBMC because whole blood RNA was not available from the TRuTH study. We previously showed that diagnostic performance of the 11-gene ACS COR signature for TB disease was equivalent in RNA from whole blood and PBMC, even though signature scores from PBMC were overall significantly lower (Darboe et al., 2018).

Longitudinal kinetics of signature scores were highly heterogeneous, although it is noteworthy that in a number of progressors and non-progressors signature scores were high at TRuTH enrolment and subsequently decreased during the study (Figure 2C). No marked differences in signature scores between progressors and non-progressors were apparent when aligned according to time after study enrolment (Figure 2C) or time to recurrent TB diagnosis (Figure 2D). Analysis of prognostic performance by ROC AUC showed weak but statistically significant (AUC 0.63, 95% CI 0.55–0.72, *p* = 0.003) differentiation between progressors and non-progressors using samples within 1 year of recurrence (Figure 2E). When further stratified into 6-month windows, statistically significant prognostic performance was only observed within 6 months of TB diagnosis (AUC 0.68, 95% CI 0.57–0.79, *p* = 0.005); not in the 6–12 months before window (AUC 0.58, 95% CI 0.45–0.71, *p* = 0.23; Figure 2F). Interestingly, when samples in the 6-month period immediately preceding recurrent TB were further stratified into 3-month windows, only the samples collected within 3 months of recurrence allowed stratification (AUC 0.72, 95% CI 0.58–0.85, *p* = 0.004; Figure 2G). These data suggest that in ART-treated HIV-infected individuals with a previous history of TB, prognostic performance of the 11-gene ACS COR signature for recurrent TB was generally limited and only allowed differentiation between progressors and non-progressors very proximally to TB diagnosis. Since investigation for recurrent TB entailed 3-monthly induced sputum it is likely that many cases were diagnosed during subclinical stages of disease. Indeed, 25 of the 43 recurrent cases (58%) were asymptomatic at the time of microbiological diagnosis.

To put these findings into perspective, we benchmarked them against the WHO target product profile (TPP) for a TB risk test (World Health Organization [WHO], 2017a), which should ideally be able to predict progression to TB within 2 years and provide a quantitative result that correlates with risk of progression. Such a test should have a minimal specificity of ≥75% and sensitivity ≥75% (World Health Organization [WHO], 2017a). At a specificity of 75%, the sensitivity within 90 days of recurrent TB diagnosis was 60% and thus did not meet the minimum criteria in the TPP (Table 3).

**Table 3:** Prognostic performance of the COR signature for recurrent TB disease in HIV-infected persons on ART, based on the TRuTH cohort.

Time to diagnosis	Sensitivity	Specificity	Number needed to screen^∗^	False positives	False negatives
WHO TPP (within 2 years of diagnosis), minimum characteristics	>75%	>75%	134	33	1
ACS COR signature 0–360 days	50%	75%	100	25	1
0–180 days	55%	75%	91	23	1
0–90 days	60%	75%	84	21	1


*TTP, target product profile (World Health Organization [WHO], 2017a). ^∗^Number needed to screen to detect one case of TB. For these calculations a TB prevalence of 2% was used. False positives and negatives were calculated per number screened to detect one case of TB.*

### Utility of the 11-Gene ACS COR Signature for TB Treatment Monitoring in HIV-Infected Individuals

**FIGURE 3:**
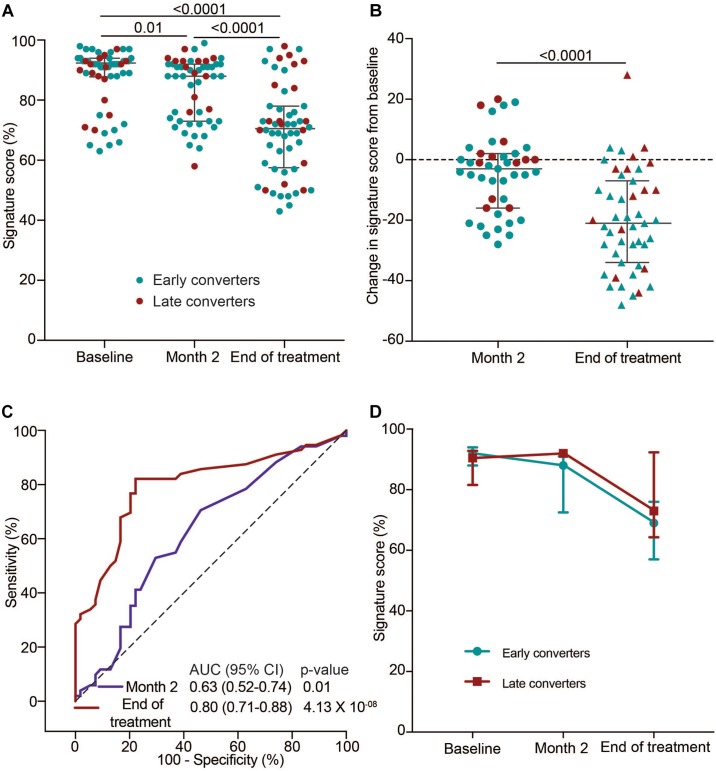
TB treatment response monitoring in the IMPRESS cohort using the 11-gene ACS COR signature. **(A)** 11-gene ACS COR signature scores before (baseline), during (after intensive phase, at 2 months) or after treatment of recurrent TB in the IMPRESS study. **(B)** Differences in signature scores at 2 months or end of treatment relative to treatment baseline. Red dots represent individuals with sputum culture conversion after 2 months (late converters) and blue dots individuals with culture conversion before 2 months (early converters). Horizontal lines represent medians and error bars the inter-quartile ranges (IQR). *p*-values were calculated using the Wilcoxon matched pairs test. **(C)** ROC AUCs depicting signature discrimination between baseline and month two or end of treatment samples. **(D)** Longitudinal kinetics of median signature scores during TB treatment in early and late converters. Error bars represent the IQR.

Next, we set out to determine if the 11-gene ACS COR signature tracks host changes during TB treatment in HIV-infected TB patients on ART with a history of previous TB using samples from the IMPRESS cohort. Since the ACS COR signature detects ISG expression we hypothesized that signature scores would decrease by the end of TB treatment to reflect cure, as shown previously in HIV-uninfected TB patients (Thompson et al., 2017). Signature scores at TB diagnosis were high in the majority of cases and decreased significantly at the end of the intensive phase, at month two (Figure 3A). This was also evident from a negative change in ACS COR signature score between these two timepoints in 62.8% of individuals (Figure 3B). Discrimination of the TB treatment initiation and month two samples using the COR signature was possible, but not impressive (AUC 0.63, 95% CI 0.52–0.74; Figure 3C). By the end of treatment most cases had even lower scores, indicating an overall reduction in Type I/II IFN inflammation (Figure 3A,B). This was also reflected by better discrimination between TB treatment initiation and end of treatment samples (AUC 0.80, 95% CI 0.71–0.88; Figure 3C). We next determined if patients could be classified into early and late converters based on their culture conversion status at 2 months of treatment. No marked differences were observed in kinetics of median signature scores during treatment between early and late converters, suggesting that the observed decrease in COR signature scores after treatment initiation was not an accurate predictor of early culture conversion in this study cohort (Figure 3D).

### ACS COR Signature, Sputum Conversion and Time to Culture Positivity

**FIGURE 4:**
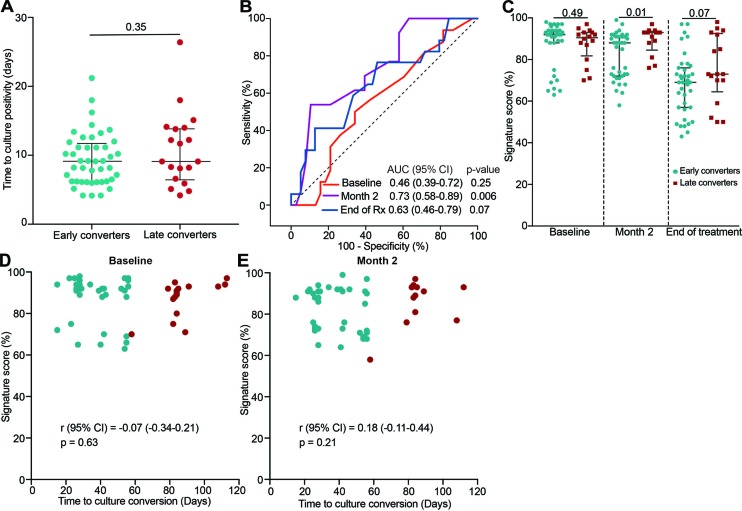
Classification of late and early sputum culture converters in the IMPRESS cohort. **(A)** Pre-treatment time to MGIT culture positivity for IMPRESS participants with sputum culture conversion before 2 months (early converters) or with sputum culture conversion after 2 months (late converters). **(B)** ROC AUCs depicting 11-gene ACS COR signature discrimination between early and late sputum culture converters at baseline, month two or end of treatment. **(C)** 11-gene ACS COR signature scores for early and late sputum culture converters at baseline, month two or end of treatment. *p*-values were calculated using the Mann-Whitney U test. **(D,E)** Association between signature scores and time to liquid culture positivity at baseline **(D)** and 2 months after treatment start **(E)**.

Next, we wanted to establish if the COR signature could differentiate early and late converters before or during TB treatment in the IMPRESS cohort. We first determined if time to sputum culture positivity at treatment baseline, a measure of bacterial load, was associated with early culture conversion (at 2 months). No difference in time to culture positivity between early and late converters was observed at TB diagnosis (Figure 4A). The 11-gene ACS COR signature, measured at treatment baseline, also could not predict early conversion (AUC 0.46, 95% CI 0.39–0.72; Figure 4B); signature scores were also not different (*p* = 0.49; Figure 4C). However, at the end of the intensive phase of treatment (month two) the signature could differentiate between early and late converters (AUC 0.73, 95% CI 0.58–0.89; Figure 4B,C). This ability was lost by the end of TB treatment (AUC 0.63, 95% CI 0.46–0.79; Figure 4B,C). Furthermore, time to culture conversion was not correlated with signature scores at treatment baseline (Figure 4D) and month two of TB treatment (Figure 4E).

It was noted that signature scores remained relatively high at the end of treatment, when all individuals had achieved clinical cure. Markedly higher signature scores were also observed in cured HIV-uninfected TB patients than matched healthy controls in the previously published Catalysis for Health Foundation (CHF) study (Thompson et al., 2017). We therefore sought to determine if gene expression would decrease further after clinical cure on the basis that residual inflammation may resolve over time. A comparison of signature scores at the end of treatment and a time point at least 6 months later showed no significant changes (Supplementary Figures S2A,B); late and early converters were also not different at these late follow-up time points (Supplementary Figures S2C,D).

### Effect of HIV on Diagnostic Performance of the 11-Gene ACS COR Signature

**FIGURE 5:**
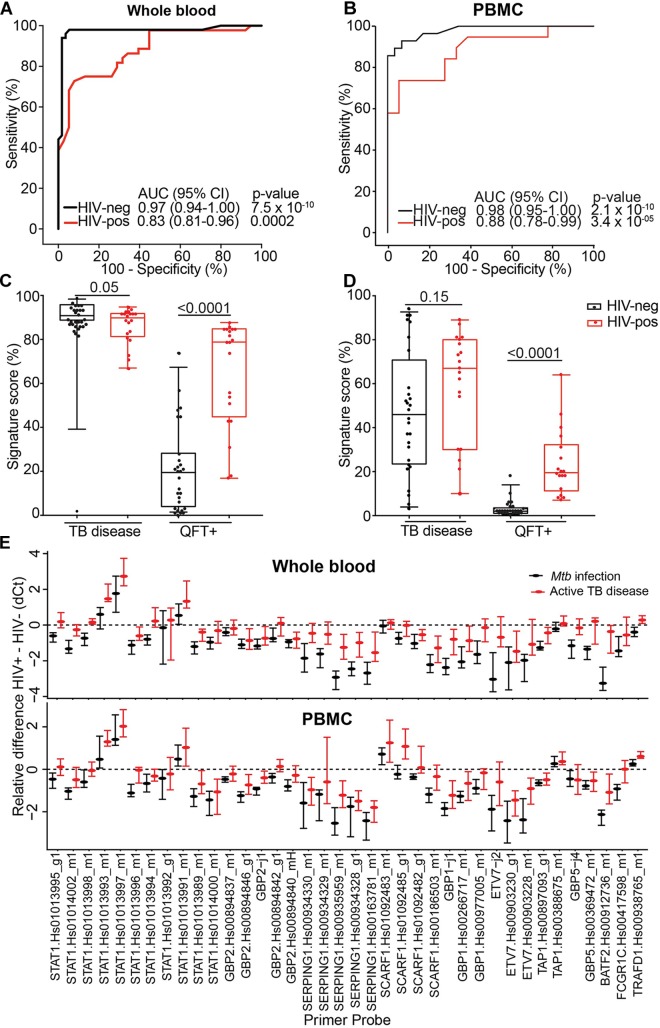
Effect of HIV on diagnostic performance of 11-gene ACS COR signature. **(A,B)** ROC curves depicting 11-gene ACS COR signature discrimination between Xpert MTB/RIF+ TB cases and QFT+ *Mtb*-infected controls in HIV-uninfected (black lines) and HIV-infected (red lines) diagnostic performance of the signature when measured from whole blood **(A)** or PBMC **(B)**. **(C,D)** 11-gene ACS COR signature scores from HIV-infected (red) and uninfected (black) TB cases (TB disease) or *Mtb*-infected controls (QFT+), when measured in whole blood **(C)** or PBMC **(D)**. Horizontal lines represent medians, boxes represent the IQR and whiskers represent ranges. **(E)** Differences in expression of individual transcripts comprising the 11-gene ACS COR signature between HIV-uninfected and HIV-infected individuals, stratified into TB cases (red) and *Mtb*-infected controls (black), when measured in whole blood (top) or PBMC (bottom). Transcripts are identified by their TaqMan primer set reference (see Supplementary Appendix A1). Dots represent medians and error bars 95% CI for each transcript, computed from delta-Ct (qRT-PCR cycle threshold) values with the rank inversion method and bootstrapping 2000 times. Negative differences indicate higher expression in HIV-infected individuals relative to HIV-uninfected individuals. We considered transcripts for which the 95% CI bounds do not overlap with zero (dashed horizontal line) to be significantly different.

Prognostic performance of the 11-gene ACS COR signature in the TRuTH study was poorer than expected from the previous studies in HIV-uninfected cohorts (Zak et al., 2016; Darboe et al., 2018; Suliman et al., 2018). Similarly, the observed reduction in signature score during treatment in the IMPRESS trial was less marked than was observed in the HIV-uninfected CHF study (Thompson et al., 2017). In light of these results and the well-established evidence that HIV infection is associated with elevated levels of plasma type I IFN protein and expression levels of ISGs in peripheral blood cells (Bosinger and Utay, 2015), we hypothesized that specificity of the ISG-based ACS COR signature would be lower in HIV-infected persons due to higher signature scores. To address this, we compared the diagnostic performance of the signature for discriminating between TB cases and *Mtb*-infected controls in HIV-infected (20 cases and 20 controls) and HIV-uninfected (30 cases and 30 controls) adults from the CTBC. Diagnostic performance in HIV-uninfected persons was excellent; AUCs of 0.97 (95% CI 0.94–1) and 0.98 (95% CI 0.95–1) were observed when whole blood or PBMC samples were tested, respectively (Figure 5A,B). However, in HIV-infected persons a striking reduction in AUC values was observed (whole blood AUC 0.83, 95% CI 0.81–0.96; PBMC AUC 0.88, 95% CI 0.78–0.99; Figure 5A,B). To understand these figures within the context of a desired triage test for TB, we benchmarked these against the WHO TPP for a triage test (World Health Organization [WHO], 2014). At a specificity of 80%, the COR signature achieved a sensitivity of 100% in HIV-negative and of 65% in HIV-infected persons (Table 4). These figures suggest that fewer HIV-infected people would have to be screened with the COR signature to detect a TB case compared with the current methods of symptom screening or symptom screening plus chest radiography (Getahun et al., 2011; Table 4).

**Table 4:** Performance of the COR signature as a triage test in HIV-infected and uninfected persons based on results from our diagnostic cohort.

HIV status	Sensitivity	Specificity	Number needed to screen^∗^	False positives	False negatives
WHO TTP for Triage test, optimal characteristics	>95%	>80%	<55	<11	1
ACS COR signature HIV-uninfected	100%	80%	50	10	0
ACS COR signature HIV-infected	65%	80%	77	16	1
**Performance of current screening tools in HIV-infected persons**					
Symptom screening only^∗∗^	79%	50%	64	32	1
Symptom screening and chest radiography^∗∗^	91%	39%	55	33	1


*TTP-Target product profile (World Health Organization [WHO], 2014). ^∗^Number needed to screen to detect one case of TB. For these calculations a TB prevalence of 2% was used. False positives and negatives were calculated per number screened to detect one case of TB. ^∗∗^Estimates for symptom screening and chest radiography are based on results from Getahun et al. (2011).*

Comparison of the quantitative signature scores showed a highly significant increase in HIV-infected relative to HIV-uninfected *Mtb*-infected controls (but not TB cases), suggesting markedly elevated ISG-expression due to underlying HIV infection (Figure 5C,D). To determine if this is driven by a subset of mRNA transcripts in the signature, we computed the median and 95% CI of the difference in individual transcript expression between HIV-infected and uninfected persons. Expression of most transcripts was significantly elevated in HIV-infected individuals relative to HIV-uninfected individuals (indicated by a negative difference in delta Ct value), irrespective of TB status or RNA source (Figure 5D,E), confirming that HIV infection elevated ISG expression. Interestingly, some STAT1 and SCARF1 transcripts were expressed at lower levels in HIV-infected than in HIV-uninfected persons, opening the possibility that a signature with more tolerance for HIV could be developed.

**FIGURE 6:**
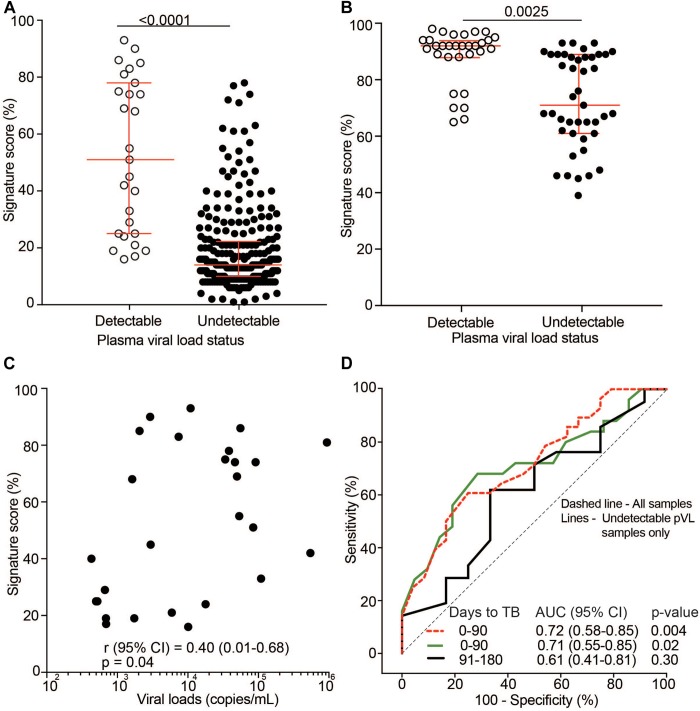
Effect of HIV plasma viral load (pVL) on 11-gene ACS COR signature scores. **(A)** 11-gene ACS COR signature scores in samples from the TRuTH cohort stratified by detectable pVL (>400 copies/mL, *n* = 27) and undetectable pVL (<400 copies/mL, *n* = 253). Horizontal lines depict medians and error bars IQRs. **(B)** 11-gene ACS COR signature scores in samples from the IMPRESS cohort stratified by detectable pVL (*n* = 36) and undetectable pVL (*n* = 50). **(C)** Spearman correlation analysis between signature scores and quantitative pVL for samples with detectable pVL in the TRuTH cohort (*n* = 27). **(D)** ROC AUC depicting prognostic performance of the 11-gene ACS COR signature for discriminating between progressors and non-progressors from the TRuTH study when considering all samples in the depicted time windows or only samples with undetectable pVL.

Next, we sought to investigate the role of HIV plasma viral load (pVL) on the transcriptomic ACS COR signature. In light of our results above, we hypothesized that signature scores are associated with pVL. Analysis of pVL as a continuous variable was complicated by the fact that most participants in the TRuTH and IMPRESS studies were on long-term ART and had suppressed pVL. Regardless, pVL were detectable at some time points and stratification of signature scores by detectable (>400 copies/mL) and undetectable (<400 copies/mL) pVL showed significantly elevated scores for samples with detectable pVL in the TRuTH (Figure 6A) and IMPRESS (Figure 6B) cohorts. In the TRuTH cohort ACS COR signature scores were also weakly associated with quantitative levels of pVL > 400 copies/mL (Spearman ρ = 0.40, *p* = 0.04; Figure 6C).

Finally, we determined if prognostic signature performance in the TRuTH cohort was influenced by pVL. Only 27 samples had detectable pVL, precluding analysis of signature performance in this subset. We therefore compared prognostic performance in all samples within 6 months of recurrent TB diagnosis or only those in this time window with undetectable pVL. Prognostic performance of the signature for recurrent TB disease was equivalent when all samples or only those with undetectable pVL were included (0–3 months before TB, AUC of 0.72 (95% CI 0.58–0.85) for all and AUC 0.71 (95% CI 0.55–0.85) for pVL <400 copies/mL; Figure 6D).

## Discussion

Blood transcriptomic signatures of TB risk show promise as non-sputum triage tests for TB, identification of individuals with incipient or subclinical disease who are at high risk of developing active TB disease (prognostic performance) and as tools for monitoring treatment outcome. However, prognostic performance of risk signatures in HIV-infected individuals who are at very high risk of TB, has not been specifically and comprehensively assessed. In the present study we determined the prognostic performance for incident recurrent TB, diagnostic performance and utility for TB treatment monitoring of the 11-gene transcriptomic ACS COR signature in HIV-infected individuals on ART.

We found that among HIV-infected individuals on ART the transcriptomic COR signature had limited prognostic value and only significantly differentiated between recurrent TB progressors and non-progressing controls within 3 months of disease diagnosis. This is markedly inferior to prognostic performance in the HIV-uninfected adolescent study, where the ACS COR signature differentiated significantly between progressors and non-progressors 1–1.5 years before TB diagnosis (Zak et al., 2016). These results, as well as broader transcriptomic, proteomic and cellular analyses of the adolescent progressors (Scriba et al., 2017), suggest that the ACS COR signature detects ISG expression during incipient and/or subclinical disease, many months before symptom manifestation. Since investigation for TB in the TRuTH cohort was based on sputum induction at every visit on every participant, we propose that the resultant early detection of disease during subclinical stages may have impeded the prognostic performance within the confines of the TRuTH study. Indeed, 58% of the 43 recurrent cases were asymptomatic at the time of diagnosis. Interestingly, a number of these asymptomatic cases (*n* = 25) refused TB treatment and subsequently developed TB symptoms. Detection of subclinical disease is not unexpected since prevalence of asymptomatic TB, defined as disease without clinical TB related symptoms but with abnormalities that can be detected with radiologic or microbiological tests, is generally high in studies that perform active case finding in high-risk populations (Drain et al., 2018). For example, in a review of 12 national TB prevalence surveys in Asia, 40–79% of microbiologically-confirmed TB cases were asymptomatic (Onozaki et al., 2015). In addition, there is some evidence that HIV-infected persons may progress more rapidly to TB disease than HIV-uninfected persons (Fitzgerald et al., 2000; Mallory et al., 2000; Charalambous et al., 2008; Drain et al., 2018), which may also account for the reduced prognostic performance observed in the TRuTH study. Despite this limited performance of the signature and low sensitivities at the specificity cut-off target of >75% in the WHO TPP (World Health Organization [WHO], 2017a), the number of people needed to screen to detect a TB case still fell below the minimum target, while also yielding fewer false positives than the target (Table 3). By contrast, tests like the AlereLAM and the new generation FujiLAM are unlikely to have prognostic utility, and application in HIV co-infected individuals beyond those with dramatically low CD4 T cell counts remains to be investigated (Maclean et al., 2019). One other study, by Sloot et al. (2015) reported a 2-gene signature comprising IL-13 and AIRE that correlated with TB disease risk in a small cohort of 15 HIV-infected drug users who developed active TB disease and 16 who did not develop TB. Unfortunately this 2-gene signature was not externally validated and thus the true performance of this highly parsimonious signature in an external cohort is currently unknown.

Provision of isoniazid prophylaxis to HIV-infected individuals typically decreases the risk of progression to active TB disease, but studies in endemic settings have shown that TB incidence increases shortly after cessation of isoniazid prophylaxis (Golub et al., 2015; Samandari et al., 2015). In the TRuTH study 20 progressors and 37 non-progressors received 6 months of isoniazid during follow-up (Maharaj et al., 2017). We did not adjust the time-at-risk in our analyses to account for effects on INH prophylaxis, but it was noteworthy that most progressors who developed recurrent TB disease were diagnosed within a few months (median of 279 days) of stopping INH prophylaxis (Maharaj et al., 2017). The effect of isoniazid prophylaxis may also have contributed to the poor prognostic performance of the ACS COR signature. Taken together, these findings highlight the importance of clear guidelines on appropriate screening and treatment strategies for latent infection and subclinical disease in HIV-infected persons.

We and others have shown that transcriptomic signatures may be useful to monitor treatment outcome in HIV-uninfected persons (Berry et al., 2010; Bloom et al., 2013; Sweeney et al., 2016; Thompson et al., 2017). In the present study we show that transcriptomic COR signature scores decreased significantly during TB treatment in IMPRESS patients, all of whom had clinical cure. However, signature discrimination between those with sputum culture conversion at 2 months of treatment was only possible at the 2-month time point, not at treatment initiation or end of treatment. It was noteworthy that cured patients retained high signature scores, consistent with elevated type I/II IFN responses up to 8 months after the end of TB treatment in the IMPRESS cohort. This finding is consistent with the observation of high transcriptomic COR signature scores at the end of TB treatment and up to a year after successful cure in the HIV-uninfected Catalysis for Health Foundation study participants (Thompson et al., 2017). These data, supported by persistent pulmonary inflammation detected on PET-CT and detection of mycobacterial RNA in bronchoalveolar lavage samples, are suggestive of residual disease or mycobacterial survival after successful cure (Malherbe et al., 2016). However, underlying inflammation resulting from HIV infection as well as low-level HIV replication, also likely contributed to elevated signature scores in the IMPRESS patients. Since the IMPRESS trial was performed in a high *Mtb*-transmission setting we cannot rule out that reinfection after cure may also have contributed to elevation of signature scores.

Taken together, our findings show that diagnostic, prognostic and treatment monitoring utility of the 11-gene transcriptomic COR signature were reduced in these HIV-infected cohorts compared with studies in HIV-uninfected individuals. Our study suggests an approximately 10% reduction in diagnostic performance in HIV-infected compared to HIV-uninfected persons, although the sample size of our study limits the strength of this finding. Nevertheless, this is in line with other studies, which also report reduced diagnostic performance of largely ISG-based transcriptomic signatures for TB disease in HIV-infected populations, relative to HIV-uninfected persons (Kaforou et al., 2013; Dawany et al., 2014; Sweeney et al., 2016; Walter et al., 2016; Zak et al., 2016). Our results suggest that the reduction in signature performance in the HIV-infected cohort is attributable to higher signature scores in those without incipient, subclinical or active disease. Regardless, diagnostic performance was still superior to symptom screening alone or symptom screening in parallel with chest radiography in HIV-infected persons (Getahun et al., 2011). Several other studies have also reported diagnostic performance of transcriptomic signatures in HIV-infected cohorts, including a large signature based on 251 transcripts (Dawany et al., 2014) and another one based on the single gene, BATF2 (Roe et al., 2016). We were not able to perform direct comparisons of the performance of the our 11-gene transcriptomic COR signature with these because this would have to be done in the same cohort. This highlights the importance of directly comparing the multiple published transcriptomic signatures directly in diverse cohorts. Notably, a very recent study performed such a systematic comparison of the diagnostic performance of 16 published transcriptomic signatures in 24 publicly available microarray or RNA-sequencing datasets and reported that a 3-gene signature performed best (Warsinske et al., 2019). It will be important to perform prospective clinical validation of such signatures in future studies and also to assess prognostic performance of these signatures for incident TB.

We also showed that detectable pVLs were associated with elevated transcriptomic COR signature scores and expression of many individual ISG transcripts comprising the signature were higher in HIV-infected compared with HIV-uninfected individuals. This is consistent with well-established evidence that HIV infection is associated with elevated levels of plasma type I IFN protein and ISG expression in peripheral blood cells (Bosinger and Utay, 2015). Our data suggest that transcriptomic signatures for TB that are discovered or parameterized in HIV-infected cohorts may yield better diagnostic performance in HIV-infected individuals than signatures developed in HIV-uninfected populations. Signatures developed by multi cohort analyses that included HIV-infected and uninfected individuals provide clues that such signatures may be more tolerant to the effects of underlying HIV infection [11, Duffy et al., under review]. Alternatively, transcripts known to be elevated in HIV-infected individuals, such as ISGs, may have to be explicitly excluded from transcriptomic signatures for HIV-infected individuals. Such a strategy was employed by Esmail et al. (2018) who identified a signature of subclinical TB in HIV-infected individuals, which comprised transcripts representing the classical complement pathway and FcγR signaling. Such a signature might equally perform well in HIV-uninfected persons. A single, “universal” signature for both HIV-infected and uninfected populations would be more feasible for implementation as a triage test in community health settings. It is not well understood if geographic, epidemiological or population genetics differences are likely to impact overall performance and utility of such a signature. The COR signature, which was derived in South African adolescents, validated with similar prognostic performance in the adult household contacts of the GC6-74 study from The Gambia and South Africa (Zak et al., 2016), although prognostic performance of the same signature in the much smaller GC6-74 test sets of Gambian and Ethiopian household contacts was not significant (Suliman et al., 2018). Notably, no modification of the COR signature was made in our study when applying it as a diagnostic, prognostic or treatment monitoring tool. The demonstrated application to cryopreserved PBMC allows broader opportunities for validation in non-African cohorts in the future, if such biobanks are available.

Our analyses were limited by relatively small cohort sizes and were not sufficiently powered to investigate signature performance as a function of pVL quantitative levels. Regardless, our stratification by detectable and undetectable pVL clearly showed a strong effect on signature scores and represents, to the best of our knowledge, a first attempt at delineating effects of HIV load on TB transcriptomic signatures. We acknowledge that a pVL assay with lower limit of detection may have allowed greater ability to interrogate the effects of VL below 400 copies/mL on the COR signature.

We also acknowledge that healthy individuals with latent infection are not the ideal control group for establishing diagnostic performance of a triage test. Future studies that assess diagnostic performance should also include control groups of symptomatic individuals who do not have TB but present with other respiratory diseases. We did not have access to such control “other disease” participants in our study.

## Conclusion

In conclusion, the transcriptomic ACS COR signature showed good diagnostic performance for TB disease, modest prognostic performance for recurrent TB and modest utility for TB treatment monitoring in HIV-infected individuals on ART. Our results suggest that underlying HIV infection has a marked effect on performance of ISG-based transcriptomic signatures, which requires further investigation in larger studies. Prospective studies of the diagnostic and prognostic performance of the transcriptomic ACS COR signature in both HIV-uninfected and HIV-infected persons is currently underway at five clinical sites in South Africa (ClinicalTrials.gov, Identifier: NCT02735590).

## Member of the Satvi Clinical Immunology Team

South African Tuberculosis Vaccine Initiative, Institute of Infectious Disease and Molecular Medicine and Division of Immunology and Department of Pathology, University of Cape Town, Cape Town, South Africa: Sindile Matiwane, Lungisa Jaxa, Noncedo Xoyana, Constance Schreuder, Janelle Botes, Hadn Africa, Lebohang Makhethe, Marcia Steyn, Onke Nombida, Rodney Raphela and Mzwandile Erasmus.

## Data Availability

All datasets generated for this study are included in the manuscript and/or the Supplementary Files.

## Author Contributions

AP-N, KN, NP, MH, DZ, and TS conceived the study. KN, GW, NP, MH, SK, DZ, AP-N, and TS raised funds and provided the resources. KN, EF, MvR, LL, NP, and MH performed clinical field work. FD, SKM, NY-Z, LL, ET, FJD, MF, NB, SM, LRM, NC, AL, GT, DG, and AP-N processed samples, performed the experiments, and analyzed the data. AP-N, KN, NP, MH, DZ, and TS interpreted the results. FD, SKM, AP-N, and TS wrote the manuscript. All authors have read and approved the manuscript.

## Conflict of Interest Statement

AP-N, DZ, ET, and TS report pending patent of the gene signature. DZ, GT, GW, and TS report receiving grants from the South African Medical Research Council, National Institutes of Health and/or Bill and Melinda Gates Foundation related to the gene signature during course of study. The remaining authors declare that the research was conducted in the absence of any commercial or financial relationships that could be construed as a potential conflict of interest.

## Supplementary Material

FIGURE S1 Clinical and demographic characteristics of TRuTH study participants at primary TB treatment in the SAPiT or START studies **(A–C)**, end of primary TB treatment **(D,E)** and at TRuTH enrolment **(F)**.

FIGURE S2 11-gene ACS COR signature scores after TB treatment in the IMPRESS cohort. **(A)** Comparison of signature scores at the end of treatment and 6–8 months later in the IMPRESS cohort. Red dots represent individuals with sputum culture conversion after 2 months (late converters) and blue dots individuals with culture conversion before 2 months (early converters). Horizontal lines represent medians and error bars the IQR. *p*-values were calculated using the Mann-Whitney *U* test. **(B)** ROC AUC depicting 11-gene ACS COR signature discrimination between end treatment samples and samples collected 6–8 months later. **(C)** ROC AUC depicting 11-gene ACS COR signature discrimination between early and late sputum culture converters 6–8 months after end of TB treatment. **(D)** Signature scores for early and late converters 6–8 months after end of TB treatment. Horizontal lines represent medians and error bars the IQR and *p*-values were calculated using the Mann-Whitney *U* test.

TABLE S1 11-gene COR signature scores in CTBC participants.

TABLE S2 11-gene COR signature scores in TRUTH participants.

TABLE S3 11-gene COR signature scores in IMPRESS participants.

TABLE S4 Worksheet for entry of raw Ct values from the qRT-PCR assay.

TABLE S5 Worksheet that calculates risk scores upon input of raw Ct values.

APPENDIX A1 Genes and primer-probe IDs of transcripts in the ACS CoR 11-gene signature.
