# Supplementary material for: Detection of Tuberculosis Recurrence, Diagnosis and Treatment Response by a Blood Transcriptomic Risk Signature in HIV-Infected Persons on Antiretroviral Therapy
Source: Front Microbiol. 2019 Jun 26;10:1441. doi: 10.3389/fmicb.2019.01441 (PMC6608601; doi:10.3389/fmicb.2019.01441)
Supplement: FIGURE S1 — Clinical and demographic characteristics of TRuTH study participants at primary TB treatment in the SAPiT or START studies (A–C), end of primary TB treatment (D,E) and at TRuTH enrolment (F). [file Data_Sheet_1.docx]

| **Gene target** | **Assay ID** | **Role in signature** |
| --- | --- | --- |
| ACTR3 | Hs01029159_g1 | Reference |
| GRK2 | Hs01056345_g1 | Reference |
| CDC42 | Hs03044122_g1 | Reference |
| CSDE1 | Hs00918650_m1 | Reference |
| CYTIP | Hs00188734_m1 | Reference |
| TMBIM6 | Hs01012081_m1 | Reference |
| TMBIM6 | Hs00162661_m1 | Reference |
| TMBIM6 | Hs01012082_g1 | Reference |
| TPM3 | Hs01900726_g1 | Reference |
| USF2 | Hs01100994_g1 | Reference |
| BATF2 | Hs00912736_m1 | Gene of interest |
| ETV7 | j2 | Gene of interest |
| ETV7 | Hs00903230_g1 | Gene of interest |
| ETV7 | Hs00903228_m1 | Gene of interest |
| FCGR1C | Hs00417598_m1 | Gene of interest |
| GBP1 | Hs00977005_m1 | Gene of interest |
| GBP1 | j1 | Gene of interest |
| GBP1 | Hs00266717_m1 | Gene of interest |
| GBP2 | j1 | Gene of interest |
| GBP2 | Hs00894840_mH | Gene of interest |
| GBP2 | Hs00894846_g1 | Gene of interest |
| GBP2 | Hs00894837_m1 | Gene of interest |
| GBP2 | Hs00894842_g1 | Gene of interest |
| GBP5 | Hs00369472_m1 | Gene of interest |
| GBP5 | j4 | Gene of interest |
| SCARF1 | Hs01092483_m1 | Gene of interest |
| SCARF1 | Hs01092485_g1 | Gene of interest |
| SCARF1 | Hs01092482_g1 | Gene of interest |
| SCARF1 | Hs00186503_m1 | Gene of interest |
| SERPING1 | Hs00934329_m1 | Gene of interest |
| SERPING1 | Hs00935959_m1 | Gene of interest |
| SERPING1 | Hs00934328_g1 | Gene of interest |
| SERPING1 | Hs00163781_m1 | Gene of interest |
| SERPING1 | Hs00934330_m1 | Gene of interest |
| STAT1 | Hs01013998_m1 | Gene of interest |
| STAT1 | Hs01013992_g1 | Gene of interest |
| STAT1 | Hs01014000_m1 | Gene of interest |
| STAT1 | Hs01013993_m1 | Gene of interest |
| STAT1 | Hs01013997_m1 | Gene of interest |
| STAT1 | Hs01013996_m1 | Gene of interest |
| STAT1 | Hs01013994_m1 | Gene of interest |
| STAT1 | Hs01013991_m1 | Gene of interest |
| STAT1 | Hs01013989_m1 | Gene of interest |
| STAT1 | Hs01013995_g1 | Gene of interest |
| STAT1 | Hs01014002_m1 | Gene of interest |
| TAP1 | Hs00388675_m1 | Gene of interest |
| TAP1 | Hs00897093_g1 | Gene of interest |
| TRAFD1 | Hs00938765_m1 | Gene of interest |

Appendix 1


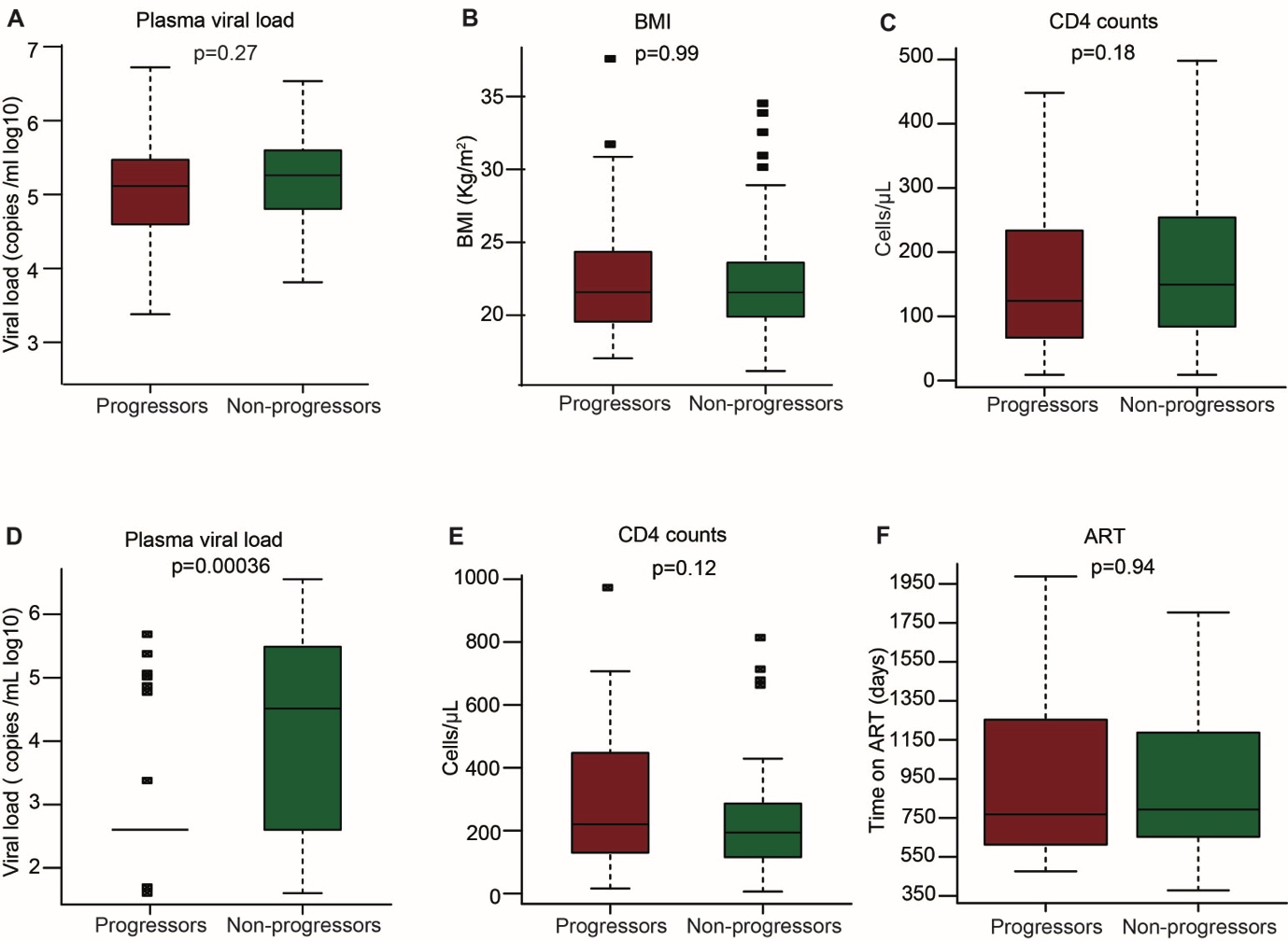


Supplementary Figure 1

Supplementary Figure 2
